# Predictors of and reasons for attempts to reduce alcohol intake: A population survey of adults in England

**DOI:** 10.1371/journal.pone.0173458

**Published:** 2017-03-09

**Authors:** Emma Beard, Jamie Brown, Eileen Kaner, Robert West, Susan Michie

**Affiliations:** 1 Research Department of Clinical, Educational and Health Psychology, University College London, London, England; 2 Department of Epidemiology and Public Health, University College London, London, England; 3 Institute of Health & Society, Newcastle University, Newcastle, England; National Institue on Drug Abuse, UNITED STATES

## Abstract

**Objective:**

This study aimed to assess the predictors among high-risk drinkers in England of attempts to reduce alcohol consumption, the reasons given for these attempts and the association between the various reasons and alcohol consumption.

**Method:**

Data came from 2,800 high-risk drinkers taking part in the Alcohol Toolkit Study (ATS) between March 2014 and November 2016 who were attempting to reduce their alcohol consumption. Participants completed the Alcohol Use Disorders Identification Test (AUDIT) and were asked questions regarding their socio-demographic characteristics, attempts to cut down and reasons for doing so.

**Results:**

Those cutting down were significantly older (OR 1.01, p<0.001), were more likely to be female (OR 1.32, p<0.05), had higher AUDIT-C scores (OR 1.12, p<0. 001), were less likely to be of white ethnicity (OR 0.64, p<0. 001), and were more likely to reside in the South of England (OR 1.34, p<0. 001). They were also more likely to be of higher occupationally-based social-grades (p<0. 001). The main reported reasons for reducing consumption were: fitness (22.5%), weight loss (20.4%), future health (20.4%), advice from a health-care professional (7.9%) and cost (7.6%). Those reporting the followings reasons for cutting down had higher AUDIT-C scores than those who did not report these reasons: a concern about further health problems (β 0.20, p<0.05), advice from a doctor/health worker (β 0.38, p<0.05), that drinking was too expensive (β 0.42, p<0.01) and detoxification (β 0.42, p<0.01). Lower AUDIT-C scores were noted among those who reported that they knew someone who was cutting down (β -0.67, p<0.05), that there was no reason (β -0.36, p<0.05), or they didn’t know why they were cutting down (β -0.25, p<0.05).

**Conclusions:**

Around a fifth of high-risk drinkers in England report trying to reduce their drinking, particularly older, high-socioeconomic female drinkers from the south of England. Attempts to cut down appear to be driven by a desire to improve health, advice from others and cutting down on the cost of drinking.

## Introduction

High-risk levels of drinking are associated with a number of social and health outcomes around the world, including an increased risk of mortality, disability and alcohol-related diseases [1]. Significant variation exists between countries, which is partially explained by environmental factors such as economic development, culture and the availability of alcohol [1]. The UK has amongst the highest per capita alcohol consumption of any country in the world [2, 3], with 9.1 million adults drinking at levels above recommended limits [4, 5]. Consequently, a target has been set by the Government in England to try and minimise the number of individuals drinking more than the ‘lower-risk guidelines’ [6].

Policies and interventions which help move individuals towards more moderate levels of consumption include the UK ban on the sale of alcohol ‘below cost’, which meant that the selling price of alcohol to consumers could not be lower than tax payable on the product, and the introduction of screening and brief intervention for risk drinking as part of NHS Health Checks [7–9]. Numerous studies have shown that brief advice on alcohol consumption by general practitioners and other health-care professionals is effective for addressing hazardous drinking, particularly in middle-aged men [10, 11]. Modelling studies also suggest that a below cost ban can lead to a reduction in annual consumption, alcohol-related deaths and hospitalisation; though the effects are nearly 45 times less than what could be achieved through minimum unit pricing [12].

To inform future initiatives and policies it is important to monitor the prevalence at a population level of attempts to reduce alcohol intake and the reasons for those attempts. This was one of the motivations behind the Alcohol Toolkit Study (ATS), which was initiated in March 2014. Although several large-scale representative surveys collect data on alcohol use in England, these do so generally only on an annual or less frequent basis (e.g. Health Survey for England, General Lifestyle Survey, and Adult Psychiatric Morbidity Survey), and do not provide consistent assessments of drinkers attempts to cut down [13]. The ATS fills this important gap by gathering and publishing monthly data on a wide range of alcohol related behaviours. This paper reports findings from the first 33 waves of data collection, providing up-to-date prevalence statistics on attempts to cut down among the general population in England, correlates of those attempts, the self-reported reasons for reducing alcohol intake

Online surveys in England have revealed that a significant proportion of drinkers (40–60%) are motivated to reduce their alcohol intake [14, 15]. Although such findings should be interpreted with caution due to the self-selective nature of participants, national surveys tend to find similar results. For example, the National Survey of Health and Development followed-up a cohort of 3,854 individuals in 1989, noting that 39.3% of women and 42.1% of men thought they ought to cut down on their alcohol intake [16]. Attempts to reduce intake appear to be even more common among sub-populations. For example, the Infant Feeding Survey in 2010 reported that 98% of pregnant women had given up or cut down on the amount they drank [17]. Up-to-date representative statistics will help in the evaluation of national and local alcohol policies and will be of interest to the UK government who are currently forming their new alcohol control strategy [18]. Such statistics will also enable comparisons with other countries. For example, around 20% of drinkers in France [19] and 40% of drinkers in Australia [20] report that they wish to cut down.

It is also important to identify the sociodemographic correlates of attempts to restrict intake. Successfully shifting the behaviour of drinkers in the desired direction (i.e. towards less harmful consumption) can be facilitated by first identifying potential explanatory variables that might impact on alcohol use. Identifying those individuals who are the most and least receptive to behaviour change will also aid policy makers trying to prioritise the spending of limited funds on public health initiatives. The decision may be to target those who are currently less motivated to change their behaviour, perhaps through screening and brief advice, as well as other approaches including online support and digital interventions [21]. Research suggests that although some individuals are able to reduce their alcohol intake on their own, those which do seek treatment either favour face-to-face support or more discrete forms of treatment due to accessibility and concerns about stigmatisation [22–24].

Previous studies have found significant socio-demographic disparities in attempts to cut down, with reductions in intake more likely among married men of non-white ethnicity, who are heavier drinkers and from lower socio-economic (SES) backgrounds [25, 26]. At the same time, other studies have pointed to a higher prevalence among younger females with more severe drug problems [27, 28]. The increase in alcohol harm reduction with age may be explained by Winick’s (1962) ‘maturing out’ hypothesis, which states that most drug use declines as individuals get older due to a stabilisation in emotions [29]. A variety of explanations have been put forward for gender differences, including the possibility of differential appraisals of alcohol-related symptoms, which may affect the interpretation of drinking problems [30, 31], and societal attitudes and social norms [32].

It is also important to ascertain the reasons or motives for attempting to cut down. This may help inform policy makers as to what factor should be incorporated into existing interventions and as to which novel policies may be the most fruitful avenues for consideration. For example, if expense is reported to promote alcohol harm reduction, this would support initiatives, such as minimum unit pricing (MUP), which aim to increase the overall cost [33]. Ill health is generally one of the main reasons given for attempts to cut down, particularly if related to alcohol intake [34, 35]. However, diagnosis of an alcohol related disorder which is symptomless may not be a potent trigger [36]. The next most commonly reported reasons include trying to avoid drink driving, to save money and to prevent injury (41%) [37]. For women in particular, weight-loss and restricting calorie intake appear to promote abstinence [38]. However, for some individuals reductions in alcohol consumption are triggered not by negative events, but a period of self-reflection; perhaps stimulated by knowing someone else who has decreased their consumption [39]. Pressure from loved ones and health-care professionals may also be important triggers [40]. In a recent survey, 78% of participants reported that they would cut down if their doctor advised them to and 66% if they were asked to do so by a partner [41]. There is also evidence to suggest that life changes and significant events (for example, a 30^th^ birthday or marriage), lead to positive role changes and consequently reduced alcohol intake [42].

There are a number of limitations with these studies to date. First, the majority have been qualitative in design and/or have had small sample sizes. Secondly, participants have been unrepresentative with a focus mainly on treated populations. Thirdly, many of the studies to date have assessed reasons for wanting to reduce intake rather than the reasons for actual attempts. It is possible that an individual may cite health and cost as reasons for wanting to cut down, but that they may be finally prompted by an acute illness or advice from a general practitioner. Finally, they did not consider the association with actual consumption levels. It is of interest to ascertain whether certain reasons for cutting down are associated with alcohol intake in order to identify which interventions the most dependent drinkers may be receptive to.

In summary, this paper aims to ascertain:

1. The prevalence of attempts to reduce alcohol consumption in a representative sample of English adults
2. Associated characteristics of attempts to reduce alcohol consumption
3. The reasons for those attempts to cut down
4. The association between these reasons and alcohol consumption

## Methods

### Design

Data were used from the Alcohol Toolkit Study (ATS) between March 2014 and November 2016. The ATS involves monthly cross-sectional household computer-assisted interviews, conducted by Ipsos Mori of approximately 1,700 adults aged 16+ and over in England. The baseline survey uses a type of random location sampling, which is a hybrid between random probability and simple quota sampling. For more details see www.alcoholinengland.info or the published protocol [13].

### Measures

All participants were asked questions about their socio-demographic characteristics. This included their age, gender, ethnicity and social-grade. Social-grade was measured using the National Readership Survey social-grades system: A: higher managerial, administrative or professional; B: intermediate managerial, administrative or professional; C1: supervisory or clerical and junior managerial administrative or professional; C2: skilled manual workers; D: Semi and unskilled manual workers; E: Causal or lowest grade workers, pensioners and others who depend on the welfare state for their income [43].

Data on government office region (North East, North West, Yorkshire and the Humber, East Midlands, West Midlands, East of England, London, South East, South West) were also collected and used to divide participants into those residing in the North and South of England. Established longitude cut offs were adopted (North: North East, North West, Yorkshire and the Humber, East Midlands, West Midlands; South: East of England, London, South East, South West) [44], which produces a boundary between the Wash and the Seven Estuary, which is similar to that suggested by historical data [45].

Participants were then asked to complete the Alcohol Use Disorders Identification Test (AUDIT) [46]. The AUDIT identifies people who could be classed as dependent, harmful or hazardous drinkers, and has demonstrated validity, high internal consistency and good test-retest reliability across gender, age and cultures [47–50]. The AUDIT consists of 10 questions: questions 1–3 deal with alcohol consumption (AUDIT-C), 4–6 with alcohol dependence and 7–10 with alcohol-related harm.

Those scoring greater than or equal to 8 on the AUDIT or greater than or equal to 5 on questions 1–3 of the AUDIT (i.e. AUDIT-C) were then asked additional questions regarding attempts to reduce their intake and reasons for reducing their intake. These are pre-defined accepted standard cut-off points for high-risk drinking i.e., hazardous, harmful and possible dependence [51, 52]. These additional questions were as follows:

1. Are you currently trying to restrict your alcohol consumption e.g. by drinking less, choosing lower strength alcohol or using smaller glasses?
  Yes
  No
2. Which of the following, if any, do you think contributed to you making the most recent attempt to restrict your alcohol consumption?
  Advice from a doctor\health worker
  Government TV\radio\press advert
  A decision that drinking was too expensive
  I knew someone else who was cutting down
  Health problems I had at the time
  A concern about future health problems
  Something said by family\friends\children
  A significant birthday or event
  Improve my fitness
  Help with weight loss
  Detox (e.g., dry January)
  Other (please specify)

Participants could choose more than one response.

### Analysis

All analyses were conducted in R version 3.1.2. Descriptive statistics are reported using unweighted data and weighted data for important prevalence statistics. Descriptive statistics on socio-demographic and alcohol characteristics are given for high-risk drinkers overall and as a function of attempts to restrict consumption. Generalised Linear Models (GLM), specifying the “Quasibinomial” family, were used to assess the association between attempts to reduce intake and socio-demographic and smoking characteristics. Both unadjusted and fully adjusted models are reported.

Descriptive statistics are also given for the reasons for current attempts to reduce intake at baseline. Further unplanned analyses were also run to assess the association between the given reasons and baseline AUDIT-C scores. Both unadjusted and adjusted models are reported.

### Ethical approval

Ethical approval for the Smoking Toolkit Study (STS), a sister survey to the Alcohol Toolkit Study (ATS), was originally granted by the UCL Ethics Committee (ID 0498/001). Approval for the ATS was granted by the same committee as an extension of the STS. The data are not collected by UCL and are anonymised when received by UCL. Explicit verbal agreement and willingness to answer questions voluntarily is recorded electronically by Ipsos Mori, the company administering the survey. This is standard protocol and agreed by the UCL ethics committee. Participants are also given a printed information sheet.

## Results

Data were collected on 55,580 individual participants between March 2014 and November 2016. Sixty-nine per cent (95%CI 68.4 to 69.2; n = 38,258) reported that they drank alcohol (unweighted: 66.1%;95%CI 65.7 to 66.5; n = 36,729); of which, 27.0% (95%CI 26.6 to 27.4; n = 15,018) were classed as high-risk (unweighted: 25.5%;95%CI 25.1 to 25.9; n = 14,171). These high-risk drinkers formed the sample for this paper. Twenty per cent (95%CI 19.1 to 20.4; n = 2800) of high-risk drinkers reported that they were currently attempting to reduce their alcohol intake.

Table 1 shows the characteristics of participants overall and as a function of whether they were cutting down. After adjustment, those cutting down were significantly older, had higher AUDIT-C scores, were more likely to be female, were less likely to be of white ethnicity, had higher odds of being social-grade AB relative to C2 to E and of living in the South of England, compared to those not cutting down.

**Table 1:** Characteristics of participants overall and as a function of whether they were cutting down.

	All high-risk drinkers (n = 14,171)	Not cutting down (n = 11371)	Cutting down (n = 2800)	Unadjusted		Fully adjustedβ	
				OR^a^	95%CI	OR	95%CI
**Age M(SD)**	44.5 (17.91)	44.0 (18.26)	46.6 (16.28)	1.01***	1.01 to 1.01	1.01***	1.01 to 1.01
**Gender %(n)**							
Male	64.7 (9174)	65.6 (7446)	61.7 (1728)	Reference			
Female	35.3 (4997)	34.5 (3925)	38.3 (1072)	1.18***	1.08 to 1.28	1.32***	1.21 to 1.44
**Social-grade %(n)**							
AB	27.3 (3874)	25.9 (2949)	33.0 (925)	Reference		Reference	
C1	34.5 (4890)	34.4 (3907)	35.1 (983)	0.80***	0.72 to 0.89	0.84***	0.76 to 0.94
C2	20.1 (2849)	21.2 (2412)	15.6 (437)	0.58***	0.51 to 0.65	0.62***	0.54 to 0.70
D	10.5 (1493)	10.9 (1239)	9.1 (254)	0.65***	0.56 to 0.76	0.70***	0.60 to 0.82
E	7.6 (1065)	7.6 (864)	7.2 (201)	0.74***	0.62 to 0.88	0.77**	0.65 to 0.92
**Ethnicity %(n)**							
Non-white	4.2 (649)	3.9 (485)	5.4 (164)	Reference		Reference	
White	95.8 (13522)	96.1 (10886)	94.6 (2636)	0.72***	0.60 to 0.87	0.64***	0.53 to 0.78
**AUDIT score M(SD)**	8.5 (3.94)	8.1 (3.61)	9.9 (4.84)	1.10***	1.09 to 1.11		
**AUDIT-C Score M(SD)**	6.8 (1)	6.7 (1.82)	7.0 (1.92)	1.09***	1.07 to 1.12	1.12***	1.10 to 1.15
**Region %(n)**							
North	58.6 (8354)	60.4 (6873)	52.8 (1481)	Reference		Reference	
South	41.4 (5817)	39.6 (4498)	47.2 (1319)	1.36***	1.25 to 1.48	1.34***	1.23 to 1.46

Note.

^a^ Unstandardized coefficients reported for all variables except age where standardized coefficients are reported to reflect the OR for a one standard deviation change in the outcome; SD = standard deviation; M = mean; % = percentage; n = number of participants.

***significant at p<0.001.

** significant at p<0.01.

β Adjusted for AUDIT-C and not the full AUDIT.

Table 2 shows the prevalence of various reasons given by those currently attempting to restrict their intake and the association with AUDIT-C scores at baseline. The top five most common reasons given were to improve fitness, weight loss, future health problems, advice from a health-care professional and cost. In adjusted analysis, those reporting a concern about further health problems, advice from a doctor/health worker, that drinking was too expensive and detoxification as reasons for cutting down had higher AUDIT-C scores than those who did not report these reasons; while those who reported that they knew someone who was cutting down or that there was no reason/they didn’t know had lower AUDIT-C scores.

**Table 2:** Reasons given for attempts to reduce alcohol intake at baseline and the association with AUDIT-C scores at baseline.

	Prevalence (n = 2800)		AUDIT-C Score		Unadjusted		Adjusted for all other stated reasons	
	%	(n)	M	SD	B	95%CI	B	95%CI
**Improve my fitness**								
Yes	22.5	630	7.1	1.82	Ref			
No	77.5	2170	7	1.95	0.06	-0.11 to 0.23	-0.08	-0.27 to 0.10
**Help with weight loss**								
Yes	20.4	572	7.1	1.83	Ref			
No	79.6	2228	7	1.94	0.12	-0.16 to 0.29	0	-0.19 to 0.19
**A concern about future health problems**								
Yes	20.4	571	7.3	2.05	Ref			
**No**	79.6	2229	7	1.87	0.34***	0.17 to 0.52	0.20*	0.02 to 0.39
**Advice from a doctor\health worker**								
Yes	7.9	222	7.5	2.33	Ref			
No	92.1	2578	7	1.88	0.5***	0.24 to 0.77	0.38**	0.11 to 0.65
**A decision that drinking was too expensive**								
Yes	7.6	213	7.5	2.12	Ref			
No	92.4	2587	7	1.9	0.52***	0.25 to 0.79	0.42**	0.14 to 0.69
**Health problems I had at the time**								
Yes	7.8	217	7.4	2.4	Ref			
No	92.2	2583	7.1	1.87	0.38**	0.12 to 0.65	0.21	-0.06 to 0.48
**Something said by family\friends\children**								
Yes	6	168	7.4	2.44	Ref			
No	94	2632	7	1.88	0.38*	0.08 to 0.68	0.22	-0.08 to 0.53
**Detox (e.g. dry January)**								
Yes	6	169	7.5	1.96	Ref			
No	94	2631	7	1.91	0.46**	0.16 to 0.76	0.42**	0.11 to 0.72
Other								
Yes	3.9	110	7.2	2.04	Ref			
No	96.1	2690	7	1.92	0.21	-0.16 to 0.58	0.24	-0.1t to 0.62
**Government TV\radio\press advert**								
Yes	3	84	7.1	1.94	Ref			
No	97	2716	7	1.92	0.02	-0.40 to 0.43	-0.07	-0.49 to 0.34
**I knew someone else who was cutting down**								
Yes	1.9	52	6.7	1.95	Ref			
No	98.1	2748	7	1.92	-0.38	-0.91 to 0.15	-0.67*	-1.2 to -0.13
**A significant birthday or event**								
Yes	1	28	7.2	1.87	Ref			
No	99	2772	7	1.92	0.17	-0.54 to 0.89	0.00	-0.71 to 0.71
**Nothing**								
Yes	4.4	123	6.6	1.75	Ref			
No	95.6	2677	7.1	1.93	-0.45*	-0.79 to -0.10	-0.36*	-0.72 to 0.00
**Don’t know**								
Yes	21	588	6.7	1.72	Ref			
No	79	2212	7.1	1.96	-0.39***	-0.56 to -0.21	-0.24*	-0.44 to -0.03

Note: participants could choose more than one reason.

***significant at p<0.001.

** significant at p<0.01.

*significant at p<0.05.

## Discussion

This study aimed to assess the prevalence and socio-demographic predictors of self-reported attempts to reduce alcohol intake in a population sample of adults from England, the reasons given for attempting to cut down and the association between the various reasons and alcohol consumption.

Just over 1/5^th^ of high-risk drinkers were found to be attempting to restrict their alcohol consumption. Attempts were more likely among older females who scored more highly on the AUDIT, and who were of a higher social-grade. The most common reasons given for attempting to cut down were to improve fitness, weight loss, future health problems, cost and advice from a health-care professional. Those who noted that the reasons for cutting down were concerns about future health, that drinking was too expensive, advice from a doctor or healthcare worker and detoxification, had higher consumption scores at baseline compared to those not giving these reasons; while those reporting that they knew someone who was cutting down, that there was no reason and that they didn’t know why they were cutting down had lower consumption scores.

Previous studies have similarly reported that around 20–40% of drinkers are attempting or want to restrict their alcohol consumption at any one time [14, 15]. It is perhaps unsurprising that older females who scored higher on the AUDIT seemed more motivated to change their drinking behaviour. Previous studies have found that women are often more concerned about their health, have greater vigilance for health-related problems and are more likely to seek help from health care professionals than men [30]. Social cognition models argue that the severity of a condition is an important motivator of behaviour change [53]; and numerous studies have shown linkages between increased severity and motivation to cut down [27]. At the same time, the findings raise concerns regarding social-inequalities in health, with lower SES individuals being less likely to report attempts to restrict their intake. This may partially explain the Alcohol Harm Paradox, the phenomenon whereby those of lower SES experience greater harm from a similar alcohol consumption than those of higher SES [54].

The importance of health related issues as a stimulus for drinking reduction is an often forgotten but well-established finding in qualitative and longitudinal studies [34, 35]. Reporting cost, fitness and weight-loss as significant motivation for reducing alcohol consumption suggests that policies targeting cost and/or affordability [33] and placing calorie information on alcohol products might be helpful levers for change [55]. It is perhaps interesting that high-risk drinkers were more likely to report *future* health-problems as a reason for attempting to restrict their consumption, given that poor *current* health is more consistently reported as a stimulus for behaviour change across behavioural domains [56, 57]. The finding that recommendations to reduce alcohol intake from a health-care professional was a significant reason for an attempt at harm reduction, supports the provision of brief advice in primary care and other health settings as recommended by NICE (PH24). Brief interventions in primary care are a cost-effective way to help patients reduce excessive alcohol consumption [10], but in practice such advice is often not delivered [11]. There are several reasons for this, including a lack of financial incentives, that GPs do not see it as their responsibility and/or do not feel confident in delivering such advice due to a lack of training.

Concerns about health and encouragement by a health care professional were more likely to be given as reasons for cutting down among high risk drinkers with greater consumption. This finding is consistent with previous studies showing that health-care professionals and doctors are more likely to encourage the most dependent drinkers to reduce their intake [11]. All those included in this study were classified as ‘high-risk’ drinkers but those with comparatively lower consumption were more likely to report ‘don’t know’ or ‘no reason’ for cutting down. It could be that health-care professionals and family members are failing to identify their alcohol problems or that they have yet to experience health implications from their drinking [11]. It may also be the case the drinkers choose to ignore advice as it is not meaningful to them or believe that alcohol consumption is a normalised behaviour not associated with impaired health [34].

This study has several advantages, including its large representative sample and assessment of a wide range of reasons and correlates. However, there are also several limitations. First, the self-reported nature of data collection may have been biased by inaccurate recall. Though there is no reason to believe that recall would differ across the various demographic groups. Secondly, due to the sample size limitations it was not possible to assess whether reasons for cutting down differed as a function of socio-demographic characteristics. As data accumulates for the ATS this will become possible, as will prospective analyses using 6-month follow-up data. Nonetheless, these findings help to identify those policies and interventions for which drinkers are likely to be the most receptive, and those groups for which specific targeting may be required. Finally, due to the cross-sectional nature of this study, it is not possible to draw causal conclusions regarding the association between the specified reasons and consumption levels. Although it is possible that many of the reasons given could ultimately lead to reductions in alcohol consumption, such reductions may be precluded by the fact that drinkers giving these reasons start at a higher alcohol intake.

## Conclusion

Around 1/5th of high-risk drinkers in England report that they are trying to reduce their alcohol intake, particularly older, high-socioeconomic female drinkers from the south of England. Attempts to cut down appear to be driven mostly by a desire to improve overall health, advice from others and cost.

## Abbreviations

ATS: Alcohol Toolkit Study
AUDIT: Alcohol Use Disorders Identification Test
SES: Socio-economic status
